# Abridged Extracts and Gleanings from Our Exchanges

**Published:** 1871-11

**Authors:** 


### PART II.

### Abridged Extracts and Gleanings from our Exchanges

*MULTUM IN PARVO.*

HEMORRHOIDS.—Dr. John H. Packard, of Philadelphia, in an article on the above disease, says: "That one of the greatest conveniences in the examination of the rectum or any of the orifices of the body, is the forehead mirror used by the laryngocopist. It obviates the annoyance of the surgeon having to dodge the shadow of his own head. While his hands are left free, he can, by changes of his head, throw the light just where he wants it, notwithstanding the movements of the patient; can be used at night or in a dark room.

Various kinds of speculums are mentioned, the surgeon using that which pleases him best. In the male subject the disease may be brought completely into view by straining over warm water so as to protrude it; in the female, by the simple plan now to be mentioned: by passing the finger into the vagina, and then turning it backward so as to hook it over the upper edge of the spincter ani, the rectal mucous membrane may be pushed down and everted so as to expose it for one or two inches. This idea is one of Dr. Storer's, of Boston. Advantage may sometimes be gained by the use of both fore-fingers, left in the anus and the right in the vagina, in estimating the actual bulk and extent of the hæmorrhagial tumors, etc. In the male we must, of course, be satisfied with the introduction of the finger into the rectum, which ought, in my opinion, always to be done in addition to the other investigating methods. Among the causes of hæmorrhoids, too little stress has been laid upon hereditary title.

*Symptoms.*—Another point which ought to have attracted more attention is the amount of sympathetic uterine symptoms which may be developed hæmorrhoids. Great distress may be induced by the grasping of piles by the spincter, although there is no protrusion whatever· This symptom is much less troublesome in cases where the spincter has become lax, and prolapsus ani is of constant occurrence ; hence it is a feature of the earlier and more manageable stage of the complaint.

*Treatment.*—In some cases, as in pregnant women, the trouble is due to a temporary cause, and will be relieved by its cessation. In others the rectal lession has lasted so long, and has become so aggravated, with relaxation of the parts, that, especially in persons past the middle age, it is better to resort to palliative measures only. In other cases, again, and above all, where a strong hereditary tendency exists, a radical and most palpable cure can be effected by judicious surgery.

One principle should govern us in all the palliative measures adopted in any case of piles, namely, to prevent straining. This may be carried out in various ways. Besides proper and nutritious food, there are four points to be attended to. By means of medicine we keep the bowels easily moved ; sulphur with molasses or small doses of epsom salts, etc., etc., will do this. The second measure is mechanical ; an opening about five inches wide by fourteen long, is made in a board, to place over the privy-seat, which allows the nates to bulge down too much, this will, in a great degree, prevent the protrusion of the relaxed rectum. The third is the use of astringent suppositories, to be used after each stool. The fourth measure is the employment of a hemispherical block of ivory or vulcanized rubber, about as large as half a billiard-ball, attached to a spring of properly adjusted strength, and this fastened to a belt. When in place it supports the parts, and prevents the descent in walking.

*Operative Measures.*—Internal piles may almost always be safely and effectually removed by ligation, and external by the knife or scissors. The cases which give the most trouble are those in which the two forms are combined.—*N. Y. Med. Journal.*

Spermatorrhœa.—Dr. R. M. Jones recommend belladonna in cases of this description where there is a loss of tone and an irritable state of the generative organs.—*Med. Record.*

Chloral is coming into use for this disease.—*Ibid.*

Chronic Diarrhœa.—Dr. E. L. Shurley, of Manistee, Michigan, has had good success in the use of the compound solution of iodine (Lugol's). He gives five drops in half a tumblerful of water, four times daily.—*Buffalo Medical and Surgical Journal.*

The external application of iodine, applied with sweet oil, has seemed to have a marked effect in many cases of chronic diarrhœa that had for a long time resisted internal treatment.—*Western Medical Advance.*

Arseniate of Strychnia, an Antidote to the Poison of Snakes.—Dr. Louis Landsyweert, (Pacific Medical and Surgical Journal,) says: "I am enabled to offer for your consideration a new therapeutic agent (arseniate of Strychnia), as an antidote to the bite of the rattlesnake. I make no pretensions to any wonderful discovery in medical science; but having, in the carrying out of the ideas I have long entertained upon this subject, through a protracted series of experiments, become possessed of certain important facts, I consider it a duty I owe to myself and your honorable body to make known the results.

We find reported in Braithwaite (xxviii. p. 423(, that five cases of snake-bite occurring in the island of St. Lucia, were successfully treated by Mr. Ireland, by the use of grain-doses of arsenious acid, in the form of Fowler's solution, given every half-hour until the patient began to revive. The number of doses varied from six to eight, which always produced copious vomiting and purging —results of the highest importance to the treatment.

In order to obtain a more readily soluble substance than arsenious acid, I made the following preparation, as an arseniate of strychnia: Dissolve thirty grains of pure strychnia in four ounces of distilled water, containing seventy-five grains of arsenic acid, and evaporate the solution until crystallization takes place, draining the crystals, and reserving the liquid, with the addition of eight ounces of alcohol, for external application. For internal use, one grain of this arseniate of strychnia was mixed with ten grains of sugar and twenty-five grains of turmeric, and divided into twelve powders—one powder administered dry, or mixed with a table-spoonful or two of wine or brandy, at the time of the bite; the same to be repeated every fifteen or twenty minutes after the first dose, in cases of the recurrence of the swelling, accompanied with pain and throbbing, the dose may be repeated in an hour. Not only in my own case, but in four others in this city, has this preparation proved in the highest degree efficacious. In order to effect a cure, in one case only have I administered more than six powders in twenty-four hours.

A New Styptic.—Collodion, 100 parts; carbolic acid, 10 parts; tannin (Pelouse's), 6 parts; benzoic acid (from gum), 5 parts. Mix the ingredients in the order above written, and agitate until perfect solution is effected. This preparation has a brown color, and leaves on evaporation, a strongly adherent pellicle. It instantly coagulates blood, forming a consistent clot, and a wound rapidly cicatrizes under its protection.—*Carlo Parvesi.—American Journal of Dental Science.*

Dr. West on Chorea.—In the Lancet of June 10th we find an abstract of Dr. West's recent lectures on disorders of the nervous system in childhood, from which we quote the following remarks:

There are two forms of chorea over which mental and moral culture have a most salutary influence. In two classes of cases it is inapplicable—those of partial chorea, and those in which movements are very violent. In the latter cases splints on the legs and arms, and swathing the body completely in a soft bandage, will often, by restraining the movements, greatly diminish the resulting fatigue. But between these two there is a large intermediate class, over which moral culture and regulated movements exercise a remarkable influence. A great drawback to success is the difficulty of arousing the will to activity. Hence gymnastics are of service in proportion to age, and are more useful when practised in a class, than when carried on alone. Music, or a simple chant, in time with which the movements can be made, helps to fix the attention and expedite the cure. Of "specific" remedies Dr. West has been able to find none which seemed to him to possess such a character. The whole class of antispasmodics—henbane, belladonna, conium, chloral, bromide of potassium—had no other effect than to cause sleep. Tartar-emetic in large doses seemed of considerable service in some severe cases with dry skin. Strychnine had not seemed to him to merit the high enconiums Trousseau passed upon it, and its use is not free from danger. The only remedy really useful, and that only in exceptional cases, is the sulphate of zinc, given in increasing doses from one to twenty grains. It had effected considerable good in some cases in which other remedies had failed, and in which there was no distinct indication for the use of this or that remedy.—*New York Med. Jour.*

Infantile Hydrocephalus.—Dr. Hugh Carmichael has written a pamphlet on this subject. His object is to show that the disease has too often been improperly treated, from the too prevalent idea that it depends on inflammation. This idea he forcibly combats, holding that it is far more often due simply to irritation, and will yield to calmatives judiciously administered. He relates cases in which children apparently moribund have recovered under calmatives, such as digitalis, cherry-laurel, etc. In some cases he has employed opium.—*Med. and Surg. Reporter.*

Dr. E. Andrews, of Chicago, recently cured a varicose ulcer of fifteen years' standing, by injecting into the veins the tincture of the chloride of iron. He afterward dressed the ulcer with carbolated cerate—carbolic acid, grs. xviii to adipis, oz. i.

Treatment of Vesico-vaginal Fistula.—Dr. G. Torres, of Turin, reports, in the Gaceta Med. de Granada, a case of cure of vesico-vaginal fistula by the postural treatment suggested by Giordano, which consists in placing the patient in a position that shall prevent an accumulation of urine in that part of the bladder in which the fistula is seated. This position is, for the majority of cases, a horizontal one with the external plane of the body. Dr. Torres cites a case in which a single cauterization with nitrate of silver, followed by the posture above alluded to, produced the closure of a fistula of one centimetre in extent. Prof. Giordano asserts that the posture alone will at times suffice to bring about a closure of vesico-vaginal fistulas. The case alluded to by Dr. Torres had existed only for about six weeks, and the recent date was doubtless a very favorable condition for the cure.—*New York Med. Journal.*

The Bromides in Epilepsy.—Dr. Lutz, in the Berliner Klinische Wochenschrift, 1871, No. 18, states that he has employed the bromide of potassium in ten cases of epilepsy with good results. In three patients the attacks were entirely suspended for six months, in the remainder they occurred in much greater intervals. Lutz began with 1–3 grs. *pro die*, and increased gradually up to 10–20 grs. daily. The author found especially good results from the combination of bromide of potassium with the bromide of ammonium. This seems to him a fresh proof that the bromide is the active principle. Also in nervous headache the bromide did good service. One case of enuresis nocturna was promptly cured.

Dr. F. G. Williams, Watertown, Wisconsin, recommends the following in the summer complaint of children:

R.—Syr. Rhei, . . . . . grs. xl.
Potass. Brom., . . . . . fl. oz. ii.

M. S. gtt. x to xx every one, two or three hours.

If there is much pain or restlessness he adds the sulphate of morphia.—*Chicago Medical Examiner.*

To Preserve Anatomical and Pathological Specimens.—The simplest means of preserving anatomical and pathological preparations, is the use of the following solution:

Saturated solution of alum, 100 grammes; saltpetre, 2 grammes.

The article to be preserved is immersed in the solution, when it becomes decolorized; but in a few days the color returns, when it is taken out of the solution, and kept in a saturated solution of alum and water only.—*American Journal of Pharmacy.*

Ice in the Rectum for Retention of Urine.—Dr. Cazenave, of Bordeaux, (Journal de Med. et de Chir., May, 1871,) says that during twenty years, the following simple expedient has never failed in giving relief in retention of urine. He introduces into the rectum a piece of ice, of the form of an elongated oval, and about the size of a chestnut, which he pushes up beyond the sphincters, and renews every two hours. Almost always, in an hour and a half or two hours at longest, urethral spasm ceases, a certain quantity of urine is passed, and the bladder is emptied without effort by the patient. If in rare and exceptional cases this does not take place, he again introduces pieces of ice into the rectum, and also places broken ice from the anus up to the end of the penis, until the urine flows, which it *infallibly* does. When there is difficulty in making water, occasioned by prostatic hypertrophy, the goods effects of the ice are rather longer in coming on, but almost always are produced. In short, in these circumstances (strictures and prostatic hypertrophies), the sedative effects are so well marked, thanks to the effects of the ice, that the introduction of bougies and sounds into the bladder and urethra is always rendered easy to practised surgeons, and hardly any pain is felt. In our Chronicle for May we mentioned Dr. Baillie's statement, that ice *per rectum* was invaluable in the narcosis of chloroform. We have now to add that the same mode of using the same agent has been reported on for retention.—*Med. and Surg. Rep.*

Treatment of Enlarged Tonsils by Injection of Iodine.—Dr. Rumbold, of St. Louis, Missouri, (Medical Archives) has treated successfully a number of cases of enlarged tonsils, by in-?ecting the glands, by means of a hypodermic syringe, with a solution of iodine—iodine, gr. ij.; potass. iod., scr. ij.; aquæ, oz. j. Generally, a slight inflammation followed the injection, but soon subsided. From twelve to seventeen injections—ordinarily two a week—were sufficient to reduce the gland to its normal condition. The advantage claimed for this mode of treatment was, saving the substance and function (?) of the gland.—*Cincinnati Lancet and Observer.*

Veratrum Viride in Puerperal Convulsions.—D. Colvin, M. D., writes from Clyde, N. Y., January, 1871, to the editor of the Medical Record, as follows:

*Sir*. In reading the proceeding of the New York Pathological Society, I was much pleased to see that the use of the veratrum viride in puerperal convulsions was meeting with much favor. For the past five years I have used it in many cases with better results than from any other course which I had heretofore.

But a few weeks ago I used it (not in such doses as were reported by Dr. Eearn to have been given by a homœopathist) in a case of eclampsia, where the consulting physician and myself could distinctly count the pulsations at one hundred and seventy per minute, and where no ameliorations of symptoms could be obtained with the use of chloroform and the other ordinary remedies in use for this grave malady. I gave Squibb's Fluid Extract, beginning with five drops, and increasing the dose one drop once in two hours until a decided impression was made upon the heart's action. Seven drops, at that interval, were all that was required to sufficiently diminish the pulsations to bring about the desired result.

I wish to say a word relative to the use of the same remedy in pneumonia. For eight years past I can truly say that, with the exception of an occasional Dover's powder, I have quite exclusively relied upon the veratrum in the treatment of this disease. Within the past year I have substituted the chloral hydrate for the Dover's powder, and find it answers a better purpose.

Hydrate of Chloral.—Dr. J. W. Reynolds, (Pacific Medical and Surgical Journal,) says: "I have been in the habit of prescribing this medicine dissolved in the syrup of orange-peel, as in the following formula:

R.—Chloral hydratis, . . . dr. ij.
Syr. cort. aurant., . . . . oz. i.

M. S. A tea-spoonful, in three or four table-spoonfuls water, every half-hour, till sleep is produced.

I have never observed any decomposition to take place, when prepared in this way. In fact, I have taken it myself for six weeks, from the same solution, having kept the bottle on my office-table, with nothing to protect it from the light, and nothing but a common cork to keep out the air.

There are still three or four tea-spoonfuls left, of four ounces I dissolved in the syrup nine months ago, and the solution looks as pure now as it did the day I made it. It is certainly more palatable in this form than in any other way I have used it, and, it is as liable to decompose in solution with water as some writers seem to think, the syrup is greatly to be preferred in this respect, for I am satisfied that a genuine article of chloral, dissolved in syrup of orange-peel, will not decompose in any reasonable length of time.

Local Anæsthesia.—Dr. C. D. Hadway, of Jerseyville, Ill., recommends carbolic acid for this purpose. He uses the crystal in a state of deliquescence, full strength.—*Med. Archives.*

REDUCTION OF DISLOCATIONS.—At the clinical society of London, Mr. Callender read a note on a plan of Reducing Old Dislocations at the shoulder. The method, illustrated by the history of a case, consisted in raising the elbow of the dislocated limb across the chest nearly to the level of the interclavicular notch, forcing the raised arm outward, rotating the arm in so doing, and lastly, whilst still rotating, somewhat depressing it. Practically, this plan of manipulation avoided all risk of injuring the great vessels in the axilla, as all pressure upon them was done away with.

URÆMIC DIARRHŒA.—Dr. J. M. Fothergill remarks (British Medical Journal, Nov. 20 1869), "In congestion of the kidneys, the flow through the convolute capillaries is impeded, and the excretory action of the kidneys is thus lost, and the blood becomes laden with effete products and water; thus altered in its physical properties, it flows sluggishly and stagnates in the capillaries, including those of the intestinal canal; spontaneous catharsis comes on, and the balance of the circulation is restored. In chronic renal disease, this becomes more necessary, and is frequently manifested. The inefficient action of the renal secreting cells, together with diluted, constricted, or thickened capillaries, produce frequently an impeded circulation; congestion and further impeded flow follows; the depurative action of the kidneys, for the time being, is held in abeyance; and blood-poisoning ensues."

Diarrhœa, therefore, in these renal conditions, Dr. F. considers to be of greatest service, freeing the blood from its retained effete prodcuts.

"Whenever, then, diarrhœa occurrs in a person," he says, "presenting the appearances of renal diseases, and more especially if there be albuminuria, or the symptoms of any renal congestion, it may be desirable to hesitate about arresting the alvine flux until some other channel be patent. From the known intolerance of opium in renal disease, preparations of the solanaceæ should be administered where the suffering is great. The skin should be immediately acted upon by the hot air bath, or otherwise; hot poultices, sprinkled with mustard, should be applied across the loins when the bath is over. Nutritive support should be given; and a mild diuretic of digitalis, and citrate of potassa in infusion of buchu or calumba, may be administered as soon as the kidneys are somewhat relieved. If a little flux remain, a few grains of powdered cassia or cinnamon may be prescribed, and the more powerful astringents should only be administered when the danger to life is imminent; and, of these, a mixture of sulphuric acid and infusion of logwood, is perhaps the least objectionable. But asstringents should be used warily and cautiously; absorbed into the blood, they astringe and arrest the activity of the bowels, but, at the same time, check and impede the action of the renal secreting cells, whose restored and renovated activity it is of the utmost importance to keep up. The action of the skin must be fostered, and the patient carefully protected from atmospheric changes, to which these sufferers are very sensitive; and, as soon as convenient, the patient must be given steel, and the other adjuncts to nutrition. In the first mild case which occurs, I shall feel inclined to try a combination of nitrate of potassa, nitric ether, and pernitrate of iron. Whichever plan be adopted, it is desirable, while affording the maximum amount of benefit, to eliminate, as far as possible, the elements of danger."—*Am. Jour. of Med. Scienecs.*

SAFE ANÆSTHESIS.—Dr. J. Petrie, of Liverpool, writes to the British Medical Journal: "I was among the first in this country, who put in use ether as an anæsthetic, after receiving intelligence of its successful employment in Boston, United States. I was then actively engaged as honorary surgeon in the Southern Hospital here; and I, along with my colleagues, continued the use of it, as well as of chloroform, in every operation for many years, without a single death having occurred to us from these anæsthetic agents. Indeed, up to this time, I have never witnessed a death from the inhaling of ether or chloroform, either in my private or hospital practice, although I have used them freely whenever called upon to do so. My object in this communication is merely, at present, to refer to the following passage in your article: "Is it true that out of all the enormous number of cases in which it (chloroform) has been administered to lying-in women, it has never produced any fatal accident? If so, what is the explanation of such a fact?" In a number of the Medical Times and Gazette of 1860, you will find a short letter of mine "On the Prevention of Accidents from the Inhalation of Chloroform."

Seeing the fact on record at the time I wrote, that seventy thousand parturient women had had chloroform during their labor without one death occurring among them, I felt it quite confirmatory of the opinion which I had for some time previously held, and which I still hold, that the position of the patient in the bed, or on the operating table, has everything to do with the immunity from danger in the former case, and with the constantly recurring fatality in the latter. In parturition, as a rule, the woman is placed on her left side, and she inhales the chloroform, with an unmeasured ingress of air, into the lungs, with little or no chance of its being interrupted in any way. The patient undergoing an operation is almost always placed horizontally on his back, on the operating table, and inhalations of the chloroform is usually affected by placing a piece of lint, or the corner of a towel sprinkled with chloroform, over the nose and mouth, the apparatus being occasionally lifted to admit air. When the patient is thus rendered insensible, the towel or lint is removed, and respiration may go on, but it not unfrequently happens that the tongue, by its weight, falls back, and the epiglottis at its base covers the larynx, and will totally prevent inspiration; so that, under this condition, with the air-passages filled with the vapor of chloroform, in two or three minutes the patient would inevitably die suffocated, or in an absolute state of apnœa.—*Medical and Surgical Reporter.*

TRISMUS NASCENTIUM.—Dr. T. L. Papin (Humboldt Medical Archives) treated a case thus:

R.—Chloroform, . . . . m xij.
Sp. æth. sulph., . . . . m xv.
Sodæ bicarb., . . . . gr. xij.
Syrup zingiber, . . . .
Aq. flor. aurant., . . . aa. dr. iv.

M.—S.—A teaspoonful p. r. n. to allay spasmodic action and secure rest and quiet.

The paroxysms after a while recurring, he resorted to the local application of chloroform to the spine, a small strip of cotton cloth wetted and applied to the entire length. This application, alternated with emollients when vesication occurred, occasional inhalations and mild purgatives was continued *fifteen* days, when the cure was complete.

Dr. Boiliniere cured a similar case, several years since, adminising the chloroform by enema.

FOR CUTANEOUS ERYSIPLAS and glandular affections Dr. J. W. Curran, in the Medical Press and Circular, recommends the following ointment spread on lint:

R.—Ammonii Iodidii, . . . dr. ss.
Cerat. Simp., . . . . oz. i.

The iodide of ammonium is also administered internally for similar diseases.

SULPHOCARBOLATE OF ZINC IN OTORRHŒA.—At a recent congress of German surgeons in Prague, Dr. Zaufal said that he had used solution of the sulphocarbolate of zinc in fourteen cases of otorrhœa, with satisfactory results. The strength of the solution was one or two grains to the ounce.—*Med. and Sur. Reporter*

Structure of the Glands of the Stomach.—Prof. Heidenhaim bas been recently making investigations on the structure of the gastric and peptic glands. The mucous membrane of the stomach of dogs was hardened in alcohol, then stained with carmine or aniline-blue, and examined with moderate microscopic powers. The glands are arranged singly like palisades, or in groups like the fingers of a glove, in close proximity to one another, and the orifice, neck and body in each can be distinguished. The orifice in the grouped glands resembles the hard part of the glove, several glands opening into it, just as the fingers of the glove open into the wider hand part. This is lined by columnar epithelium. The neck or narrow portion of each tube is lined by roundish colored cells. The body is lined by two kinds of cells, one external or marginal, round, and colored, the other, small, internal, and uncolored, though their nuclei sometimes become tinted. The former, Prof. Heidenhaim calls investing-cells, (belegzellen,) the smaller uncolored ones, he names chief-cells (hauptzellen). The former probably represent the peptic cells of writers. The lumen of the glands is occupied by granular dark material. He describes with full details the action of the various re-agents upon the two above mentioned forms of cells. He then gives the results of his researches on the glands during the digestion. They increase in size; the chief cells become swollen, and their contents are finely granular, showing that they have absorbed more than they have secreted. The investing cells are less altered. No division or multiplication of cells was observed.—*N. Y. Medical Journal.*

Dr. Richter uses in big warts, caustic potash repeatedly, or the crystalized carbolic acid, after the knife. Tannin, sulphuric acid, acetic acid, do not work so well.—*Zeitschrift Parasitenkunde.*

Oak-Bark Wash in Sumach Poisoning.—Dr. J. B. A. Risk, Morgan, Ky., writes (Cincinnat Medical Repertory): "In my experience in the treatment of the erysipelatoid affection of the skin and subjacent tissue induced by any one of the family of the rhus, whether the radicares, toxicodendron, vermix, etc., nothing has been so satisfactory in its curative effects as the decoctio querri albæ; indeed I regard it as a specific; for if the parts diseased are bathed in the warm decoction sufficiently, the soothing effects, the speedy subsidence of the pain, and tumefaction and redness soon follow, announcing to the sufferer the sanative influence of this agent. The subsidence of the inflammation and the corrugation of the skin will not perhaps always take place at the first bathing, but, if followed up a few times, will be sure to occur, ending in a permanent cure, without the use of constitutional remedies. In order to effect these results the parts diseased should be in contact with decoction, either by immersion or by application with a sponge for the space of thirty or forty minutes, or even longer, when there is much inflammation, and repeated every four hours." —*The Medical Cosmos.*

MONTERVERDI made some experiments with sulphate of quinine and says:

1. It is a tonic in general, and especially to the uterus.
2. Half hour after use it causes light and painless contractions, slowly increasing to actual labor-pains.
3. For expulsion of the fœtus and placenta, 35 centigrammes are sufficient.
4. It is preferable to ergot, because it is not poisonous to the mother or child; the contractions are regular and it can be given in any stage.
5. It is useful in metrorrhagia, amenorrhœa and puerperal fevers.
6. It is as good as a tonic in diseases of the digestive and genito-urinary organs.
7. Pregnancy requires caution.
8. The action can be neutralized by opium.
9. Hysteria contra indicates its use.—*La Nueva Liguria Med.*

A SIMPLE way for examing the throat is to make the patient yawn. In this way powder can be blown in the best.—*Uuion Medical.*

PROF. CATANI has cured 25 cases of diabetes mellitus. The diet was meat only, no milk or eggs, and 5 grammes of lactic acid in water daily. C. uses alkalies in acute rheumatism.—*Weiner Med. Presse.*

REDUCTION OF HERNIA IN THE ERECT POSTURE.–Dr. McGeachy, in the Canada Lancet, gives a case in which the patient was relieved by taxis in the erect posture, and adds the following remarks:

"1st. Obstinate constipation, or complete occlusion, may sometimes be caused by a partial incarceration of a portion of an intestine, which neither digital examination nor any physical means can properly denomstrate. The extreme importance of a proper diagnosis in suspected cases need not be insisted on. This patient had taken for two or three days previous enormous does of salts, but without any effect; this I was not aware of the time. I had a very interesting case of this kind sometime ago, which terminated on the fourth day in complete relief, by spontaneous reduction.

"2d. Is the erect posture the proper one, or only accidentally advantageous? Might I presume to offer a theory to my medical brethren, which, in the absence of any other that I am aware of, may be thought worthy of some consideration?

"*I believe that the proper position, theoretically, for the reductian of a strangulated ingninal hernia, and in which alone the cooperation of dynamic agencies can be utilized, is the erect posture, with the flexure and adduction of the thigh.*

"The means to be used are obvious. If beforehand the colon be well evacuted, or as much so as possible, every rational preparatory condition will have been fulfilled. In the old positions, but one force is brought to bear—the *pushing force* used by the operator, if I may so term it. By this method we have also a pulling force (*vis a fronte*), namely, the weight of a large portion of the bowl striving to drag the remainder from its posture of imprisonment. Why not, then, invert the patient, and secure the action of this new force in a still greater degree? Simply this: The rythmic action of the diaphragm forbids the continual operation of this force, and should it have any effect, it often leaves matters *in statu quo*, during its contraction. Besides the force here would generally be acting at an angle, the ring being the fixed point.

"3d. Many practical men prefer this method of reduction, without regard to theory."—*Medical and Surgical Reporter.*

Treatment of Gonorrhœa by Warm Water Injections.—Dr. John O'Reilly (Am. Practitioner), in recommending warm water injections in the treatment of gonorrhœa, says, that the subjoined conclusions may be drawn from his experience: 1st. That gonorrhœa yields to local treatment, and even water injections. 2d. That water injections or medicated lotions owe their efficiency to their frequent application. 3d. That the common small syringe should be done away with in treating this disease, and none used but those throwing a continuous stream. 4th. That large injections, by fully distending the mucous membrane of the urethra, insure a speedier cure than those less copious.—*Canada Lancet.*

Ricini Collodion (Collodium cum ol. Ricini.—This preparation, a mixture of collodion and castor-oil, is strongly recommended in cholera. The Archives Generales de Medicine, says, that thirty to forty grammes painted over the abdomen will arrest cholera in the algid stage. It stops the vomiting of cholerine and cholera, and provokes a sudoral crisis, in which the poison is eliminated.

INTERNAL HÆMORRHOIDS TREATED WITHOUT OPERATION.—During the past year, Dr. Beckman has treated at the New York Dispensary eleven cases of internal hæmorrhoids, all occuring in females, and all treated without operation. In every case, the only internal medication consisted in the employment of the following formula:

R.—Pulv. Sennæ, Potass. Bitartrat.,
Pulv. Sulphuris, . . aa. oz. ij.
Pulv. Zinziberis, . . . oz. ss.

This preparation is designated in the Dispensary Pharmacopœia, as *Pulvis Sennæ Compositus.* The dose, as employed by Dr. Beckman, was a teaspoonful of the powder in molasses, every morning. The local treatment consisted in the use of the following ointment:

R.—Ext. Belladonnæ,
Plumbi Acetatis, . . . aa. dr. ii.
Acid Tannic, . . . . oz. ss.
Ung. Adipis, q. s. et fiat unguentum.

A small mass of the ointment to be introduced within the anus thrice daily, after a thorough ablution of the parts with cold water. The duration of the treatment was quite various, bearing a direct ratio to the severity of the case, ranging from a week to about five weeks. As far as could be ascertained, recovery took place in every instance, and no case of relapse has thus far come to Dr. Beckman's notice. A few of these patients suffered from hæmorrhage, but not to an excessive amount. Instead of the ointment above mentioned, Dr. Beckman uses, in private practice, suppositories made up of the same ingredients, with the exception that cocoa-butter is substituted for the simple ointment—each suppository containing two grains each of extract of belladonna and acetate of lead, with four grains of tannin.

TREATMENT OF SUPPURATING GLANDS OF THE NECK.—Dr. Lawson Tait employs the following treatment: "It consists simply in tapping a gland as soon as it is ascertained to contain pus, and in continuring this treatment until the cavity no longer secrets. The means with which he effects this is the little instrument known as Dr. Alexander Wood's morphia syringe. Only two precautions are necessary, and these are, never to introduce the needle twice at the same spot, and to introduce it very obliquely into the abscess, entering it at least half an inch from the margin of the tumor. As a rule, the direction must be from behind, forward: but occasionally it may be done from before, backward, or at right angles to this direction. He has had to persevere till he has made as many as fifty punctures at intervals of from one to ten days before the treatment was successful; the success being constituted by the complete disappearance of the tumor without the skin ever breaking. The amount of satisfaction the patients express at being saved the disfigurement of a gland-mark amply repays in thanks the great amount of trouble required.—*British Med. Jour.*

In a note upon the above communications, in the following number of the same journal, Dr. John Murray observes that, in his opinion, more attention should be shown by practitioners to the simple plan of drainage than is customary. In the case of a girl recently under his care with several enlarged cervical glands, one of which, as large as a chesnut, had already suppurated, Dr. Murray passed at once two silk ligatures through the tumor, by means of a needle, and tied the free ends. The mother was directed to keep the openings patent by moving the ligatures a little twice or thrice a day. In a week the tumor had much diminished, a considerable quantity of pus having been discharged. He then introduced, in place of the silk sutures, one of Prof. Lister's catgut ligatures, soaked in carbolic oil, which answered the purpose admirably. This was removed in three or four days. A fortnight after the first visit the abscess had ceased discharging, and the needle wounds had healed. The part was then painted with collodion. —*Practitioner.*

Mitchel's Process for Removing External Tumors.—Wm. A. Bell, M. A., of London, gives an interesting account of the mode of operation for the removal of tumors practised by a French charlatan, for a knowledge of which Mr. Bell paid no less a sum than 25,000 francs, and which, having now obtained complete information, he has very properly and liberally made public. The preparation used in all cases where the tumor can with safety be reached externally, is made in the following way: Asbestos, as soft and free from grit as possible, is reduced by rubbing between the hands to the finest possible fleecy powder. It is then mixed thoroughly with three times its own weight of strong sulphuric acid ($S\ O_3\ H\ O$). A mass is thus formed which may be easily worked with a silver or gold spatula into any size or shape corresponding to the tumor to be destroyed. Any malignant growth of the breast which is detached and solitary, with the submaxillary glands unaffected, is suitable for treatment, whether open or not makes no differance. In the application of the caustic, the adjoining healthy parts of the skin are carefully protected by applying a zone of collodion and pads of linen, and the patient is so placed that the surface of the tumor is perfectly level. The saturated acid asbestos is then laid on the surface to the thickness of half an inch for a tumor of the size of a hen's egg. Rapid destruction of the tissue follows, with, after the first half hour or so, but little pain. An oozing of clear watery fluid appears, which must be carefully sopped up. After twelve or fourteen hours' action, the first application is to be removed, and a new portion of smaller size adapted to the sore. After this has been applied for twelve hours, the operation is complete, and the healing of the deep excavation alone requires to be attended to, for the details of which we must refer our readers to Mr. Bell's phamphlet. Mr. Bell does not pretend to say that this mode of operation will effect a permanent cure of cancerous cases, but he thinks that the plan presents various and considerable advantages over extirpation by the knife, as in producing much less shock to the system, in removing the tumor alone with but little of the surrounding breast, and in postponing, in malignant cases, for a longer period the recurrence of the disease.—*Detroit Review of Medicine and Pharmacy.*

Therapeutic Value of Gelseminum.—Gelseminum (or, as it is sometimes written, gelsemium) is of late attracting considerable attention. It is highly lauded by some practitioners as a nervous sedative, in cerebral congestion, mania, and a great variety of disturbances resulting from disorder of the nerve-centres. We know of one physician who regards it as invaluable in nervous or sick-headache; ten or fifteen drops of the tincture to be given three times daily. The physiological effects of the agent are very remarkable. Even moderate doses will sometimes produce a peculiar, heavy sensation in the forehead, with partial paralysis of the levator muscles of the eye-lid, so that it is difficult to keep the eye open. We have employed it frequently for a number of years, often with benefit, but certainly not with such happy results as some others ascribe to it. The following formula will be found valuable in hysterical and functional disturbances of the nervous system:

R.—Tinc. valeriannæ ammon., . . oz. i.

Tinc. gelsemini, . . . . dr. i.

M. Sig. A teaspoonful p. r. n.

Some of our druggists prepare an ammoniated "elixir" of valerian, which is better than the officinal tincture, in being much less disagreeable.—*Pacific Medical and Surgical Journal.*

Phantom Tumor.—Dr. J. S. Dorsey Cullen, of Richmond, Va., contributes to the Virginia Clinical Record, the following interesting case:

"The patient had, for four or five years, suffered greatly from distension of abdomen, pain in the ovarian region, great nervous disturbance, constipation, and retention of urine.

"Before entering the hospital in 1868, her symptoms simulated ovarian disease so much in the eyes of her medical attendants that the operation of ovariotomy was seriously contemplated, and was only postponed to gain her consent.

"It became evident on careful examination that the distention of the abdomen was not owing to the presence of an abdominal tumor, but was one of those phenomena seen so often in hysterical females. Its eccentric changes of form and dimension at different hours of the day, its tympanitic character, the absence of any well-defined body within its walls, its entire freedom from uterine attachment—these and many other spmptoms not necessary to eliminate in the discussion the question of uterine or ovarian disease.

"Besides tense and distended abdomen, there were other and painful symptoms which pointed to uterine, vesical, and gastric causes. There was constant nausea, with bloody and stercoraceous vomiting, intense pain over the stomach and between the scapulæ, with retention of uterine. Her nervous system with all of this was wrought to its highest pitch of excitement, so much so that the mind at times gave way under it, and she became almost demented.

"This condition of affairs went on for thirteen months or more, increasing in severity each day, until death by inanition and exhaustion put an end to her misery.

"The treatment had, whilst under the present medical officer, been directed to the relief of the gastric troubles, which seemed to stand out prominently as the chief cause of her suffering. To this end, bismuth, nitrate of silver, ice, cooling drinks, blisters, etc., were perseveringly tried. Her urine was drawn off every two or three hours by the catheter, and her bowels relieved by enemas. Concentrated and nutritious diet was given her, a little only of which could be retained.

"Autopsy showed the uterus and its appendages to be perfectly free of disease, and the abdominal cavity innocent of a tumor. The mucous membrane of the stomach was only slightly inflamed, and had no appearance of ulceration."

Prurigo Treated by Ointment of Iodoform.—Prof. Tanturri, of Naples, has used the ointment of iodoform in obstinate prurigo. This compound, first brought prominently into notice by Bouchardt, is now employed extensively, not only for glandular enlargements, but also, owing to it anæsthetic properties, in skin diseases accompanied with intense pruritis; its odor is much more agreeable than chloroform, resembling that of saffron. Moretin and Humbert recommend it for internal use as possessing all the advantages of iodine, of which it contains 90 per cent., without any of its inconvencies. It exercies upon the sphincters a local anææsthetic effect so powerful that defecation is sometimes performed unconsciously after its use; it therefore forms an admirable suppository in cases of tenesmus, hemorrhoids, etc. Moutre's formula: iodoform, powdered, gr. xx.; cocoa butter, dr. j., melt and mix for six suppositories. For frictions the ointment is used in the strength of dr. j. to the ounce of simple ointment.

A Speedy Cure for Rheumatism.—Dr. R. H. Boyd states that he cures inflammatory rheumatism in from three to seven days by the following method: He gives first a full emetic dose of ant. et potass. tart., and when this has operated, five drops of tinct. opii and five drops tinct. colchici every three or four hours, and a teaspoonful of a half-pint mixture, containing dr. iv. potass. acet. every hour. When the patient becomes very hungry, and is quite free from pain, having fasted several days, he allows two teaspoonfuls of milk or one oyster three times a day, increasing the quantity gradually each day.—*Michigan University Medical Journal, May,* 1871.

Red Ink.—Red ink, which, it is said, will not lose its beautiful bright color after hundreds of years:

R.—Best Cochineal Carmine, - - gr. iv.
Liquor Ammonia, - - - - oz. iij.
Gum Acacia, - - - - gr. x.

First put the carmine and liquor ammonia into a suitable vial; then add the gum acacia, and allow it to dissolve, when the ink will be ready for use.—*Medical and Surgical Reporter.*

Quassia for Surgical Dressings.—"Flies cannot bear the smell of the wood, maggots are therefore entirely avoided," says Mr. C. C. Mitchinson, in the Lancet. The use of an infusion of quassia as a dressing for open wounds and ulcers in hot climates, and during the prevalence of hot weather, he recommends, and states that in the United States army, after one of the James River engagements, five hundred wounded men, under the care of a friend of his, were treated in the above manner.

Ulcers of the Os Uteri—Uterine Plug.—M. Despres, Surgeon to the Paris Venereal Hospital for Females, has published a work, which is referred to in the London Lancet, recommending local treatment almost exclusively for ulcerations of the cervix uteri, and attaching equal value to differennt caustics, such as the red-hot iron and nitrate of silver. He says they all cure in about the same time. He also extols the *plug*, made as follows: Take a small piece of coarse gauze, in which a pledget of cotton-wool is placed, after it has been filled with about fifteen grains of powdered alum. Fold the gauze over the wool, and tie the ends of the former with a thread five or six inches long, which is allowed to hang out of the vigina, to facilitated the removal of the plug. The latter should remain twenty-four hours.

Chloral in Toothache.—Dr. Page, in a letter to the British Medical Journal, states, that for some time past he has employed chloral hydrate, not only as an internal sedative in dental neuralgia and caries, but also as a local application to a carious tooth. A few grains of the solid hydrate placed on a quill point introduced into the dental cavity speedily dissolved, and the pain is either deadened or effectively allayed. A second or third application of the remedy may be necessary.—*The Doctor.*

Irrigation of the Pharnyx.—Dr. Guersant (Med. News and Library) alludes to those cases of very intense inflammation of the pharnyx where the patients have much trouble in opening their mouths, and can scarcely separate the teeth. For these cases, and especially for children, in simple or violent inflammation of the tonsils, in abcess of these organs, in pseudo-membranous stomatitis, in dyphtheritic pharyngitis, or gangrenous inflammations, or even in chronic granular or other forms of angina, much advantage is derived from irrigations of the throat. As a syringe or an irrigator must always be used, it would only be a question of having a suitable canula to depress the tongue and make the fluid spirt into the different parts of the throat. The ordinary gum canulas do not depress the tongue, and may be bitten and broken by children. In order to overcome this defect, he had has constructed an instrument which is called the canula tongue-depressor, made of bronze aluminium, that metal having the advantage of not being altered by sulphurous water, which may be used under certain circumstances, in chronic amygdalitis for example. It has the shape of the extremity of a spoon-handle, is slightly curved, from five to six inches in length, and one-fifth of an inch thick, and hollow in its whole extent. Its extremity which is designed to depress the tongue, has on its circumference and on its convexity a certain number of small holes, like those of a watering-pot. At the other end is a true canular extremity, which may be adjust to the caoutchouc tube of an irrigator, or even of of syringe.

These irrigations may be repeated several times a day. Older persons easily accustom themselves to its use, and even children at last consider it an amusement. In certain forms of chronic amygdalitis, and in granular inflammation, he has obtained good results from irrigation, by the use of sulphurous waters, as practised by Dr. Lambron, at Luchon.

Dahlberg's Tincture.—Tinctur colocynthidis, known also as "Dahlberg's Tincture," is made as follows: Colocynth pulp (cut small and free from seeds), oz. i; aniseed, dr. i; proof spirit, 1 lb. Digest for eight days, express and filter. Dose, 6 to 20 drops.—*Pharm. Journ. and Transactions.*

Gangrenous Vulvitis in Children.—Dr. Guersant (Med. News and Library) publishes the following treatment of this affection. It may be local or general, the principal indication being to combat the disehse which debilitates the patient. The various kinds of tonics, as cinchona, bitter tonics, broth, wine, coffee, etc., may be placed in the first rank. The local treatment is not less indispensable. Applications of lemon-juice, powders of cinchona and camphor, vinous lotions, etc., may answer the purpose, but these means often fail. The best means of limiting the gangrene is the use of iron heated to a white heat. Cauterization should go beyond the boundary of the slough, which may still often be crossed by the disease the next day. When this powerful local agent succeeds, there remains a large ulcer, a real burn of variable depth, which requires the same dressing as burns of this region. It is important that slightly camphorated powders of cinchona and charcoal should be applied; and lotions, including aromatic wine or chlorinated water, may be found of service. Finally, all possible precautions for the prevention of adhesion of the labia are of the highest importance. The dressings should be carefully made and frequently renewed.

The Use of Iron in Scarlatina.—Russell Alridge, M. D. (British Med. Journal), is desirous of drawing the attention of the profession to the use of iron in scarlatina. He has given it for the past two years with great success, so much so as to induce him to believe that in it we have a powerful remedial agent for that disease. Not only does it shorten and lessen the severity of the attack, but it also fortifies the patient against the after-consequences—dropsy, etc. The form which he has mostly used has been the liquor of pernitrate of iron in syrup or glycerine, in doses of ten minims every three hours for children of from one to six years, increasing, according to age, to fifteen, twenty-five or thirty minims. During convalescence he has given citrate of iron and quinine, ammonio-citrate of iron, or syrup of phosphate of iron, according to circumstances.

*Capillary Bronchitis.*—In speaking of the treatment of this disease, Dr. T. M. Julian, of New York. (Medical Record) says:

The sulphate of zinc and tincture of sanguinaria have, for the last twenty-five years, been my favorites, and rarely, if ever. in the hour of trial, have they failed me, either singly or collectively, aided in some obstinate cases by the application over the whole chest, and at suitable intervals, of Stocke's liniment.

With those simple means at my command, and close watching, I do not remember in a long series of years but one or two failures. To a child, say from six to eighteen months of age, I generally administer at the beginning of the disease from one-half to one grain of the sulphate, and from five to ten drops of the tincture every two or four hours; but when called at a later period, and especially when it threatens suffocation, as much as three of the former to thirty drops of the latter, from one-half to two hours apart, regardless of vomiting. In acute cases generally from four to eight hours suffice to put the patient out of danger.

As regards the effects of the above-named remedial agents on the adult mucous membrane, the following case will serve as an illustration:

"In the afternoon of the 21st of August, 1866, I was requested to visit Dr. Steine, a German practitioner residing within a stone's throw of my office in Hoboken. Being at that time absent, I did not reach his bedside till near 10 P. M., and then found him in the following condition:—Insensible; dorsal decubitus, with shoulders propped on pillows; surface of body cold and clammy; face and extremities livid; eye fixed and insensible to light; pulse feeble and rapid; trachea and bronchial tubes full of mucus; in fine, in a state of collapse. From his numerous and distressed family surrounding, as it seemed to be, his death-bed, I gleaned the following history:—That for about ten or twelve days past he had complained of a troublesome cold, for the relief of which he had sought the advice of one of his Teutonic colleagues residing in the city of New York, whose last prescription had been the application of cold wet towels to the chest. In compliance with this suggestion he gave it during the forenoon a fair trial, but soon after seemed to get worse, till the present urgent symptoms supervened. At first, impressed with the hopelessness of his condition, I was disposed to set aside any fruitless attempt and allow the patient to depart in peace, considering the unavailing efforts previously made by two or three of the more brilliant luminaries of the Hoboken medical fraternity: but, impelled to action by a crowd of anxious and beseeching relatives and friends, and bearing in mind the old aphorism, "*melius est anceps remedium quam nullum*," I determined on a forlorn-hope trial of the effects of zinc and sanguinaria. I hastily sent for an eight-ounce mixture containing dr. iv. of the sulphate and oz. ij. of sanguinaria tincture, oz. vj. of water, and set about giving at first one-half and soon after a full tablespoonful, every half hour, of the mixture, which, on account of the torpid deglutition, I had great difficulty in getting down. I covered the patient *de novo* with warm mustard poultices, and put hot bottles about the body. By 12 M. the skin had lost a great deal of its icy and clammy coldness; pulse a shade better and more resisting; tried in vain the feather to the throat to excite vomiting, continuing the mixture as stated above. 3 A. M.—Pulse stronger, skin warmer; all at once patient made an effort as if wanting to vomit, which, aided with the feather and the lowering of his head over the bedside, brought to a successful issue the long-wished-for object, and a quantity of frothy mucus was ejected with the contents of the stomach. He was soon after able to take and keep down brandy and beef-tea. Skin became warm and moist; pulse natural; mucous rattle soon disappeared, and by 4½ A. M. he fell into a quiet and apparently refreshing sleep. Left him then, and at my next visit, 9 A. M., had the satisfaction to shake hands with him, he sitting up in bed."

*The value of the Sulphate of Iron as a Local Application in Phlegmasia Dolens.*—This method of treatment was first adopted by the author many years ago, from the great sneeess reported by Velpeau from its use locally in erysipelas. It had been employed exclusively in that form of phlegmasia dolens commencing at the calf of the leg and extending upwards to the groin, where the veins are chiefly involved. It has been applied as a lotion (twenty or thirty grains to one ounce of water), as hot as the patient could comfortably bear it, generally by means of spongio-piline. All the cases so treated had made good and rapid recoveries, contrasting favorably with cases formerly treated by leeching and hot fomentations. Muriated tincture of iron was, at the same time, given in large doses. The same method of treatment was suggested in other cares of phlebitis.—*Dr. R. W. Critchon.*

*Pneumothorax Treated by Aspiration.*—At the London Hospital, Dr. Ramskill had recently under his care two patients who suffered from pneumothorax. They show, says the Lancet, the great value of the pneumatic aspirator in the treatment of the disease.

In the case of a phthisical girl, æt twenty-eight years, the smallest needle of the aspirator was introduced, in which air was discovered in the left pleural cavity, posteriorally between two of the lower ribs, and seventy-four ounces (by measure) of air was drawn off, and the relief given instantaneous.

In the case of a male patient, æt fifty years, the right side of whose chest was resonant from the second rib, and tympanitic from the root of the lung downward, and whose liver was pushed below the ribs, the small needle was introduced at the ninth intercostal space, and between sixty and seventy ounces of air was pumped out. The patient recovered.—*Ibid.*

Notes on Chloral.—Mr. Fairthorne says, in the last number of the American Journal of Pharmacy :

"Pure hydrate of chloral, according to Dr. Rieckher, does not take fire when heated in a spoon over a spirit lamp, but evaporates without residue [the alcoholate when similarly treated inflames.] Nitric acid sp. gr. 1.20, either cold or hot, should not produce any reaction with it. I find that its aqueous solution produces a dense precipitate when mixed with solution of subacetate of lead.

The hydrate is readily dissolved by alcohol, ether, oil of turpentine, benzole, bisulphide of carbon and the fixed oils. The solution in the last named article might prove of value to the physician as a topical application, perhaps available in neuralgic or gouty affections.

When equal parts of camphor (in small pieces) and hydrate of chloral in crystals are shaken together in a vial and allowed to stand, they become fluid, forming a clear solution. This might also be of use as an external remedy.

Treatment of Scabies.—Monti (Jahrbuch der Kinderheilk., iv. 2) has tried the effects of copaiba and of carbolic acid in cases of scabies in children. Copaiba produces on the tender skin violent burning and redness ; these symptoms, however, disappear in half an hour. The itching usually dissappears after the first inunction ; and the effervescence ceases after three or four applications. The remedy has no further influence on the eczema. Better results have followed the application of carbolic acid (one drachm to a pint of water or to four ounces of lard). By the diligent rubbing of the affected part with this three times a day, the itch is usually cured in three or four days.

Treatment of Chlorosis.—M. Delioux DeSavignac proposes, in the Bulletin de Therapeutique, the following formula as being likely to fulfill most completely the ordinary indications of treatment in chlorosis: tartrate of iron and potash, ten grammes; powdered aloes, two grammes; powdered castor, two grammes; powdered saffron, one gramme; to be made into a mass with Venice turpentine and divided into a hundred pills. At first, three pills are to be given daily, and the number is to be gradually increased to six or nine; care being taken to produce a free action of the bowels without producing diarrhœa. The pills are to be taken three times daily; early in the morning, and at luncheon and dinner. M. Delioux DeSavignac explains that the aloes is intended to obviate the constipation frequently met with in chlorotic patients; the castor to relieve flatulent distention of the abdomen; while the turpentine exercises a beneficial influence on the leucorrhœa. If the aloes act too energetically, it may be replaced by rhubarb; or, if the constipation remains obstinate, a little jalap, scammony, or gamboge, may be added. If the bowels be already free, M. Delioux DeSavignac omits the purgative altogether. When the turpentine produces gastric disorder, or colic without diarrhœa, balsam of Peru may be substituted for it. The use of the pills is contra-indicated in chlorosis attended with menorrhagia.—*Medical and Surgical Reporter.*

Nitrite of Amyl.—Prof. Silliman, in a recent lecture before the Yale Medical Institute, described the nitrite of amyl as an effective agent for the removal of muscular spasm. Its vapor, he says, extinguishes flame, and acts as an anti-septic. When inhaled, it acts in a wonderful manner to excite the circulation—the action of the heart in man, and in warm-blooded animals, being doubled in rapidity in thirty seconds. This intense action is followed by great suffusion of the skin, by breathlessness like that produced by violent running, by a peculiar sensation and fullness of the head, with throbbing, and ultimately by failure of muscular power of the extremest kind. It produces no destruction of the nervous sensibility, and in animals no increase of sensibility up to the moment of death. It is not an anæsthetic. It appears to act by arresting the progress of oxidation in the tissues.

As a remedy for disease, the great virtue of nitrite of amyl is its specific power of removing muscular spasm. Beneath its first and most conspicuous power of producing excessive action of the heart and apparent excitement, there is another and more permanent condition produced, namely, a temporary paralysis of muscle and a suspension of all outward manifestations of life which in the lower animals (frogs) can be sustained without actually destroying life. The observation of Dr. Richardson led him to point out the importance of this nitrite to control spasms, and especially to meet spasmodic disease, tetanus (or locked jaw), over which he inferred it would have a direct controlling power. This sagacious suggestion has since been completely verified: first, by Dr. Brunton, of Edinburgh, in *angina pectoris*, and subsequently by others in the same disease, as also in terrible pain from spasm of the bowels, where, when the nitrite was administered, the patient exclaimed that he "was transformed from agony to heaven in a moment." Mr. Forster, an eminent practitioner of England, administered it to a man suffering from tetanus, where the spasms were so severe that the patient is described as "having been rolled up like a rigid ball." Five drops of the nitrite inhaled from a handkerchief induced immediate lessening of the spasms. This treatment was assiduously renewed on each return of the spasms for nine days, when he had inhaled an ounce of the fluid, and the case was a complete recovery.—*Medical and Surgical Reporter.*

HEMORRHAGIC SMALL-POX.—Mr. Aisken, in the hemorrhagic form of small-pox, has observed the most beneficial results from the administration of two table-spoonfuls of the following mixture every three hours:

R.—Liquoris strychniæ,
Tinct. ferri muriat, . . aa dr. j.
Inf. quassiæ, . . . . ad oz. viij.—M.

The action of the strychniæ, as a nerve tonic, is that which is chiefly sought. Mr. Aisken, as well as others of the medical attendants on small-pox in London, are much impressed with its value.

*Chloral and Bromide Potassium Combined in the Treatment of Chronic Alcoholism.*—Dr. Bradnack, in the Buffalo Medical and Surgical Journal, calls attention to what he terms an original method of treating chronic alcoholism, viz: by the "use of a combination of unusually large quantities of chloral hydrate and bromide of potassium. Thus, to a case verging on mania from use of alcohol, he gave two drachms of hydrate of chloral and eighty grains of bromide of potassium. These doses were given at 9 P. M. The patient immediately fell into a tranquil sleep, and did not wake till eleven next morning." During the rest of the day he was very drowsy, but his appetite returned, bowels moved freely, pulse was 70, skin moist. No more medicine was given. He slept well next night, and the following day was well enough to go on a business journey lasting four days.—*Detroit Review of Medicine.*

ANTI-PERIODICS.—Signor Asprea Vincent states, (Lo Sperimentale, June,) that cafeine and salicine are inferior to quinine in hemicrania. He has found sulphite of magnesia and tincture of iodine valuable anti-periodics when quinine does not succeed or is contra-indicated. In children, he uses quinine, mixed with lard, as a local application to the epigastrium. Ten or twelve times the dose by the month is required; a little rancidity of the lard assists the action. The same plaster will serve for several times. When intermittents are complicated with diseases of the stomach or bowels, sulphite of magnesia is superior to quinine. From twenty to thirty grammes, in four doses, at intervals of two hours, will arrest the paroxysms. It is just as efficacious if it produces a purgative effect. From eight to nine hours after commencing it the patients often experience itching and burning of the skin, which lasts a quarter of an hour or twenty minutes. About five cases in thirty fail to be benefited by the sulphite.

The tincture of iodine succeeds, according to Dr. Aspera Vincent, in all intermittents, except in those of children, or where there is excitement of the digestive organs, or periodic headache. He gives it in doses of four drops, increasing one drop per day up to ten or twelve drops, and relates some surprising successes. He finds relapse after this treatment does not occur. If the thoracic organs or bronchial tubes are irritable he adds a little ipecac. A single dose of four drops of tincture of iodine, early in the morning, freqently arrests the next paroxysm. It can then be given four or five hours before the expected return. It will be remembered that Willebrow drew attention (Arch. de Med.) two years ago to the febrifuge powers of iodine.—*The Doctor.*

*Treatment of Puerperal Mania.*—The first step to be taken in the treatment of puerperal mania is to protect from violence the patient, her surroundings, and especially her infant. This may be best effected by the prompt use of the strait-jacket. As soon as the patient finds herself powerless, and becomes exhausted, the paroxysms become less frequent and less severe. Freedom of motion, in a dark room, is more apt to maintain the raving. If we wait for a large strong woman to become quiet, the time required would be so long that there would be great danger of inducing local puerperal processes by such long-continued violence. Besides, a dark padded room is scarcely to be found, except in a hospital where such diseases are especially treated. During the paroxysm, purgatives, followed by two-grain dose of opium, proved effectual in restraining the same. In cases of congestion of the heart, the opium should be combined with some cathartic medicine, and leeches should be applied to the temples. If there are local inflammations they should be treated locally. In chronic cases, the use of the bromide of potash was followed by good results. The diet should be of the most nutritious character. Tepid baths were useful in quieting the paroxysm. As soon as there are any evidences of an improvement in the symptoms, *all* exciting causes, however slight, should be scrupulously avoided. As the majority of these patients are of delicate constitution, frequently chlorotic, or anæmic, iron and quinine are especially indicated. Wine or stimulants of any kind we have generally reserved until the convalescent stage. Close attention should be paid to the extraordinary appetite which is very frequently present during convalescence; for the least mistake in the diet would very like.y cause a resurn of the disease.—*Journal of Gynœcological Society.*

*Artificial Respiration in Suspended Animation.*—In Bain's method, the patient is laid upon his back on a table, and the operator, standing at the head, pulls the shoulders horizontally toward him with a certain degree of power, placing, for this purpose, the fingers of each hand in the axilla, in their front aspect, with the thumbs on the clavicles. In Pacini's method, the patient and operator are in the same relative position, but the operator takes hold of the arms of the patient behind, and close to the armpit, while the thumb is in front of the head of the humerus. He then pulls both shoulders toward him, and lifts them in a perpendicular direction, by which means the sternum is first raised by means of the clavicle, and, in consequence, the ribs, which, diminishing their obliquity to the spine, enlarge the thoracic cavity both in its tranverse and antero-posterior diameters. A committe of the Medico-Chiurgical Society, report that by either plan, as also by Dr. Sylvester's, of which they are merely modifications, a sufficiently large quantity of air is without difficulty introduced into the chest.—*Braithwaite's Retrospect.*

*A Simple Dressing for Fracture of the Clavicle.*—Dr. Lewis A. Sayre, of New York, (Am. Practitioner), has finally reduced the treatment of this fracture to *two strips of adhesive plaster, withont an axillary pad;* and as such he now gives it to the profession as the simplest and most efficacious plan yet devised.

His method of keeping the inner portion of the clavicle from riding over the outer portion, is *by putting the clavicular portion of the pectoralis major muscle on the stretch*, and compelling it to *pull* the clavicle in place, and thus overcome the tendency of the clavicular portion of the sterno-cleido-mastoid to elevate it, which it will always do unless this precaution is taken. After drawing the arm backward and retaining it there by a strip of adhesive plaster, pass another piece of plaster from the *well shoulder* across the back, and by pressing the elbow well forward and inward, the first plaster around the middle of the arm is made to act as a fulcrum, and the shoulder is necessarily carried upward, outward, and backward; and the plaster, being carried over the elbow and fore-arm (which is flexed across the chest) to the opposite shoulder, the place of starting, and then secured by pins or stitches, permanently retains the parts in position.

Dr. Sayre formerly commenced the first plaster on the inner side of the biceps; but he found that this muscle would roll around and the plaster would lose its hold, requiring to be renewed occasionally; and if it completely encircled the arm, for the purpose of a stronger attachment, it would arrest the circulation, and then prove dangerous. He uses strong and good adhesive plaster (Maw's moleskin is the best) cut into two strips, three to four inches wide (narrower for children). By this plan of treatment the patient is only detained from his daily avocation a sufficient length of time to properly adjust the strips of adhesive plaster.

*Similarity Between Nocturnal Enuresis and Epilepsy.*—Dr. J. B. Bradbury, Physician to Addenbrooke's Hospital, Cambridge, (British Medical Journal) says there is one point in connection with nocturnal enuresis which has interested him very much, and that is the close similarity between this affection and epilepsy; indeed, nocturnal enuresis might, without any great error, be called enuresis of the bladder. The points in which the analogy holds are: 1st, Enuresis and epilepsy are both decidedly hereditary, and one neurosis may be transformed into the other; patients who have had incontinence of urine in youth sometimes becoming epileptic after puberty. 2d, Both affections are influenced by the system of nerves—the sympathetic, which may, under certain circumstances, induce spasm in the muscular fibers of the small arteries of the brain, as it does in the unstriped muscular fibers of the detrusor vesicæ muscle. 3d, Belladonna is of service in the treatment of both these affections, and probably acts by its influence on the sympathetic. 4th, Epilepsy may be either essential or due to reflex irritation, and so may nocturnal enuresis

He is of opinion that hydrate of chloral will be found useful in the treatment of some forms of this affection.

